# Our Exchange Journals

**Published:** 1846-05-01

**Authors:** 


### MEDICAL INTELLIGENCE, EDITORIAL, &c.

*Our Exchange Journals.*—In our seventh number we commenced an enumeration and brief account of the Medical Journals, respectively, which we receive in exchange. We had space in that number to mention one only, the *American Journal of Medical Sciences*. It was our intention to continue the subject in the succeeding number, but owing to the press of other matter of more immediate importance the next and each successive month, it has been deferred. We will not, however, allow this volume to terminate without redeeming the promise implied in that unfinished design. We owe this much to our reaaers, deeming it, as we do, not the least of the duties of the Journalist to bear testimony to the valuable efforts of others in the same department of useful labor; and in view of the beneficial influences to be exerted by periodical literature, to endeavor to contribute to its better appreciation and support by the profession in all its instances: in so doing we discharge a pleasing duty not only to our contemporaries, but to ourselves, since we would hope to render some slight acknowledgement for the cordial and friendly encouragement with which a stranger, and unpracticed in editorial mysteries, we were welcomed as a member of the fraternity. We have already mentioned the American Journal of Medical Sciences published in Philadelphia, hitherto, if not still the Medical Paris of America. Several other Journals emanate from this city; of these we have received regularly the *Bulletin of Medical Sciences, Edited by John Bell, M. D., etc.* This is a monthly, of 35 pages. It is published in connection with the *Select Medical Library*, of which we shall take another occasion to speak. Taken singly it is afforded to subscribers at the low rate of $1,00 per annum. The editor, Dr. Bell, has been long and favorably known to the profession as a journalist, an author of several useful works, an editor of various republished publications, and especially as a co-author with Dr. Stokes of Dublin, of a standard work on practical medicine. His journal is all that from his reputation as a medical writer and collator would be pre-supposed. The other Philadelphia Journals are the *Medical Examiner*, edited by Prof. Houston, and the *Journal of Pharmacy*, edited by Drs. Carson and Bridges. Of these we can only speak from their general estimation. We believe them to be creditable and useful periodicals; and we hope that when we can claim a more equal footing as regards dimensions, they will give us an opportunity to testify to their value from a more intimate acquaintance. There are two journals devoted to Dental Science in that city which should be included in the category of medical publications. One of these, the *Journal and Library of Dental Science*—we have never seen. It is, however, we believe, edited under the direction and patronage of the college of Dentistry recently organized. The other is entitled "*Stockton's Dental Intelligencer*," edited by Stockton, the famous manufacturer of artificial teeth. It is a monthly, of 24 pages, $1 per annum.

With a wide geographical leap we pass from Philadelphia to New Orleans. Here we find a bi-monthly work of 140 pages, with typography not inferior to that of any other, having four editors, two of whom are connected with the Louisiana Medical School. It is entitled the *New Orleans Medical and Surgical Journal*, $5,00 per annum. This is one of our ablest contemporaries. A large portion of its contents consists of original matter; and whoever is desirous to be informed of the peculiarities of disease, and medical practice in that locality, will do well to become a subscriber—he will also have the satisfaction of patronising a work of sterling merit, which will return more than an equivalent for the price of subscription, apart from the particular advantages alluded to.

At Augusta, Georgia, is published the *Southern Medical and Surgical Journal*, edited by Drs. Eve and Garvin, both professors in the medical college of Georgia. It contains 64 pages, monthly, at $3,00 per annum.

The mechanical execution is excellent, and the literary and scientific character of the work in keeping. We have not been of late so familiar with this publication as we should have been glad to have been. The publisher has either forgotten us, or we must attribute to the uncertainty of the mail the disappointment of not receiving it with regularity.

*The Southern Journal of Medicine and Pharmacy*, edited by Drs. Smith and Sinkler; a bi-monthly of 120 pages, at $4,00, has recently been established at Charleston, S. C. It has reached its second number only. It bids fair to become a valuable addition to the periodical medical literature of our country.

We return now Northward: and at Louisville, Ky., we have the *Western Journal of Medicine and Surgery*: The editors are Drs. Drake, Yandell and Colescott, the two former professors in the Louisville Medical Institute. This is a monthly of 90 pages, at $5 per annum. This journal has been of many years' standing, and is a substantial, independent work, managed, as would be expected from the well known talents of its editors, with much ability. A considerable portion is devoted to reviews.

*The Western Lancet* is now published at Lexington, Ky: It was formerly published simultaneously at Cincinnati, Ohio, and at Lexington. The editor is Prof. Lawson of the Transylvania University, a rival institution to that at Louisville. It is a monthly of nearly 50 pages, at $3 per annum. It is a spirited publication, and gives proof of that peculiar taste and judgment in its editor, which qualify for the editorial task.

Passing into an adjoining state, Missouri, St. Louis furnishes two medical periodicals, as also two medical schools. *The St. Louis Medical and Surg. Journal*, edited by Drs. Linton, McPheeters and Fourgeaud, (the two former connected with the St. Louis University,) is a monthly of 40 pages, at $3. *The Missouri Med. and Surg. Journal*, edited by Drs. McDowell and Barbour, of the medical dept. of Kemper College, is likewise a monthly, containing 24 pages at $2,00 per annum. It would be invidious to distinguish either of these as possessing merits superior to the other. Were we in a humor to criticise, we doubt if we could readily find fault with either. It deserves to be chronicled as an item of evidence against the reproach so often quoted as proverbially applicable to the medical fraternity—to wit—the disposition to contention and detraction—that the two rival journals just mentioned, in the same city, and conducted by members of rival schools, uniformly maintain toward each other a courteous bearing, unmarred by even honorable disputation. Doctors surely do not always disagree; a thousand facts might be adduced to prove tho falsity of the old apothegm which conveys this slander.

From St. Louis we journey to Chicago. Here our friend and quondam colleague, Dr. Blaney, fills up the little leisure, which he can spare from his retorts and crucibles, in preparing a monthly periodical of 16 pp. at $1., entitled the *Illinois Med. and Surg. Journal*. We perceive by the last No. which we have received, that he is about to enlarge its dimensions to 90 pp., and publish it bi-monthly at $2. We heartily wish it the success it merits; it deserves distinguished success, and we doubt not will secure it.

A voyage of a thousand miles brings us to Buffalo, but here we cannot tarry; the iron horse whirls us onward a half-thousand miles, and we quickly find ourselves in the American Athens. We hasten to pay our *devoirs* to our old friend—excuse the familiar expression; we cannot boast a personal acquaintance with the editor of the *Boston Medical and Surgical Journal*, and yet we have weekly chatted with him for more than a decade of years. The Boston Journal is a weekly, or monthly, just as its patrons elect. It is the only weekly medical periodical in the country. Always punctually prompt at the appointed time, always containing some racy, sententious editorials, with items of medical news precisely what the reader wishes to know—every practitioner who has $3. per annum of his income to spare, inflicts upon himself great injustice, if be not enumerated among its numerous subscribers, who are distributed over every part of our country. The Boston Journal is one of the oldest in the Country. We do not know its precise age, but it has lasted two or three generations at least, and the decrepitude of declining years is not yet apparent.

Here, also, is a new candidate for professional and public favor,—viz.—*The Journal of Health;* W. M. Cornell, M. D. Editor; a monthly of 24pp. at $1. per annum. It has reached its third number. Its objects are legitimate and worthy, and we trust they may be properly appreciated and sustained.

But hold—in our railway speed we passed by the mammoth Asylum for the insane at Utica; the institution so honorable to the intelligence and munificent philanthropy of the Empire State. Let us return.—But we cannot now describe this charity-palace, and the admirable arrangements by which its benevolent objects are so effectively secured. Dr. Brigham, the distinguished superintendent, to whom the excellence and usefulness of the institution are chiefly due, does not content himself with bounding his good deeds by the walls of his establishment, but through the *American Journal of Insanity*, is actively and efficiently engaged in promoting and diffusing knowledge in this not least, if not the highest branch of medical science. His Journal is published quarterly at $1. per annum, 96pp. It is the first and only periodical organ of this specialty, either in this country or in Europe. It is what the merited reputation of the Editor would lead us to expect.

Imagine ourselves projected in a parabolic curve toward the north, and impinging upon one of the cities of her Brittanic Majesty's Canadian Provinces. At Montreal, from the ashes of the *Montreal Gazette*, lately sprang the *British American Journal of Medical and Physical Science*. It is conducted by Drs. Hall and Macdonnell, both lecturers in the M'Gill College. It is a folio sheet, double columns, and is issued monthly, containing 27 pp. at $3. per annum. This is a sterling work, characterised by solid and practical qualities, and a high scientific tone. We are gratified to observe an entire freedom from national prejudices in its relations with the neighbouring medical literature of our republic. This is in accordance with the truth that science and learning constitute a republic in themselves, which is independent of, and above all distinctions of caste, sect, or place.

We come now to terminate our rambles in the American Metropolis. New York has hitherto been unfortunate in her medical periodicals; those which have at successive periods been established, have been short lived: so that the ephemeral existence of any which should undertake to live there, had almost passed to a proverb in the profession. The *New York Journal of Medicine and Collateral Sciences* was established some six since, by the late Dr. Forry; under his management it was rapidly growing into the confidence and favor of the medical public, when his labors were terminated by his untimely death. For the last two years it has been in the hands of Dr. C. A Lee, well known as a highly accomplished medical scholar, practitioner and lecturer, and one of the most industrious and able medical writers of the day. Under the editorial management of Dr. Lee, this journal has not only maintained the high character it had previously acquired ; bnt we may say, without any disrespect to the memory of its founder, that it has progressively advanced in excellence. Without invidiousness it may now be compared with any other American Journal. Its stability is secured, and we have only to hope that its editor will not allow himself to be diverted from his present intention to remain permanently connected with it. This is a bi-monthly of 144 pages, at $3 per annum.

*The Medical and Surgical Reporter*, the only medical periodical in New-York city beside that just noticed, will complete our catalogue. This is a bi-weekly, containing 16 pages, for $2 per ann. Its chief object is to communicate to the profession the clinical lectures of the teachers of the two medical schools, and reports of interesting cases occurring at the hospitals. The plan is a good one; and thus far has been very satisfactorily carried out. The work must be especially useful to those who witness the cases which are the subjects of the lectures reported, and indeed, is scarcely less so to those who are deprived of this privilege, affording an approximation to the advantages which they enjoy who can observe the sensible phenomena presented in connection with the important practical preceps enunciated and enforced.

We have thus enumerated and spoken of all the Medical Journals in North America, with which we are acquainted even by title. We have bestowed upon all our humble meed of praise. We confess that we had no disposition to subject all, or any of them to a searching criticism, assuming for the moment, that we have the ability if we were so disposed. But if we could not have said what we have with a clear conscience, we assure our readers that we should have left the the whole matter untouched.

